# A spatiotemporal analysis of inequalities in life expectancy and 20 causes of mortality in sub-neighbourhoods of Metro Vancouver, British Columbia, Canada, 1990-2016

**DOI:** 10.1016/j.healthplace.2021.102692

**Published:** 2021-10-30

**Authors:** Jessica Yu, Laura Dwyer-Lindgren, James Bennett, Majid Ezzati, Paul Gustafson, Martino Tran, Michael Brauer

**Affiliations:** a School of Population of Public Health, University of British Columbia, 2206 E Mall, Vancouver, BC, V6T 1Z3, Canada; b Institute for Health Metrics and Evaluation, University of Washington, 3980 15th Ave. NE, Seattle, WA, 98195, United States; c Department of Epidemiology and Biostatistics, School of Public Health, Imperial College London, Norfolk Place, London, W2 1PG, United Kingdom; d Department of Statistics, University of British Columbia, 3182 Earth Sciences Building, 2207 Main Mall, Vancouver, BC, V6T 1Z4, Canada; e School of Community and Regional Planning, University of British Columbia, 433 - 6333 Memorial Road, Vancouver, BC, V6T 1Z2, Canada; f MRC Centre for Environment and Health, Imperial College London, London, United Kingdom; g Abdul Latif Jameel Institute for Disease and Emergency Analytics, Imperial College London, London, United Kingdom

**Keywords:** Health equity, life expectancy, cause-specific mortality, small area models, geospatial analysis, urban health

## Abstract

Spatially varying baseline data can help identify and prioritise actions directed to determinants of intra-urban health inequalities. Twenty-seven years (1990-2016) of cause-specific mortality data in British Columbia, Canada were linked to three demographic data sources. Bayesian small area estimation models were used to estimate life expectancy (LE) at birth and 20 cause-specific mortality rates by sex and year. The gaps in LE for males and females ranged from 6.9 years to 9.5 years with widening inequality in more recent years. Inequality ratios increased for almost all causes, especially for HIV/AIDS and sexually transmitted infections, maternal and neonatal disorders, and neoplasms.

## Introduction

Central to many present-day public health dilemmas is the paradox of wealth and health in urban centers (1,2). Alongside increasing opportunities for employment and access to services in cities, income, social, and population health inequalities have increased (3–5). Urbanisation has been shown to widen health inequalities and increase the number of people at the extreme ends of the disease morbidity and mortality distributions within major global cities (6). These equality issues have become global concerns with increasing urbanisation; at present more than half the world’s population lives in urban centers (4). Reducing inequality within cities is therefore a key step for improving population health (7,8). Although these concerns are acknowledged in global development and sustainability agendas, there is still an unmet demand for high resolution local data for accountability and surveillance. Specifically, spatially varying data are urgently needed to help prevent and contain the trajectories of future population health challenges (9). In the United Nations’ *A World That Counts* report (10), there are calls for greater investments in cities for comprehensive and up-to-date data to increase the capacity of local governments. The UN/DESA Policy Brief #89 also calls for a whole-of-government approach to data governance to support the collection and report of disaggregated data to identify and address special vulnerabilities and needs of vulnerable groups (11). If such data needs are left unmet and neighbourhood-level health experiences are not considered, urban inequalities may be further exacerbated.

In the wider literature, there has been little to no prior comprehensive research on historical patterns of intra-urban inequality at sub-neighbourhood spatial scales while also considering analyses of cause-specific mortality (8). With the exception of limited studies conducted in the United States (12–16), United Kingdom (17,18), and Australia (19), most previous small area analyses were restricted by data availability or high sampling variability, which requires pooling and reporting of multiple years of data especially as counts become too small for traditional life tables and analyses by cause. The limited number of small area analyses of intra-urban gaps reported up to 18.3-year gap for males in King County, USA (highest compared to lowest CTs) (20) and up to 17.7 years for females in Santiago, Chile (difference of ninth and first decile of subcity units) (21). A national-level study in England and Wales found a gap of up to 9.7 years for males (highest compared to lowest deprived districts) (17). Additionally, a study in Rotterdam found an average 8-year difference in health-adjusted life expectancy between neighbourhoods (22) and a small area study in London adjusted for disability and found more than two-folds difference among males in percent of life spent in disability between London wards (23). Intra-urban estimates in Canada are even more scarce apart from two studies in Ontario (24) and Nova Scotia (25), which quantified intra-urban premature mortality and life expectancy; the former limited the outcome to only premature mortality over a 4-year period, while the latter study pooled data over multiple years and communities with a minimum population of 5000. These limited studies suggest that there is substantial variation in life expectancy at the district, neighbourhood, and sub-neighbourhood levels within urban cities, although very few analyses evaluated the specific causes of death driving this variation.

In response, we aim to quantify sub-neighbourhood indicators of health to measure spatial heterogeneity and identify clusters of cause-specific deaths in a large metropolitan city in Canada. Recent ecological investigations of mortality in British Columbia (BC), the third most populous province in Canada, observed lower preventable premature mortality rates and a higher life expectancy by up to 10 years for the highest-ranking local health area (LHA) compared to the lowest (26). Compared to other LHAs in the province, Greater Vancouver experienced higher longevity and lower premature mortality rates. Given previous studies that have observed significant disparity in some of the healthiest counties and districts in North America (27) and Europe (17), we hypothesised that measurable within-city health disparities were present and had widened over time in major Canadian cities. Therefore, higher spatial resolution health estimates are needed to assist local-level planners to assess the underlying determinants to direct resources and services accordingly.

To address these gaps, we applied and extended the use of Bayesian small area statistical models (12) at the census tract (CT) level to a 27-year linked cause-specific mortality dataset in Metro Vancouver, Canada. By doing so, we hope to address the overarching aim of this study to identify and assess temporal patterns of intra-urban LE inequalities and drivers of these inequalities by assessing multiple causes of mortality.

## Materials and Methods

### Overview

We estimated LE at birth and cause- and sex-specific mortality for 368 census tracts (CTs) in Metro Vancouver annually from 1990 to 2016. We report temporal patterns of median life expectancy from birth by sex, changes in 20 cause-specific mortality groups for both sexes combined, 9 subset cause-specific and age-standardised mortality rates by sex in 2016, and the median, absolute inequality gaps, and relative inequality ratios of the 90^th^ percentile (P90) and 10^th^ percentile (P10) CTs of all mortality measures. This study received institutional review board approval from the University of British Columbia Behavioural Research Ethics Board (H18-00246).

### Data source

Death records of all registered deaths from January 1, 1990 to December 31, 2016 were collected from BC Vital Statistics through the Population Data BC (PopDataBC) secure research environment (28). PopDataBC coordinates a harmonised and transparent data access procedure to facilitate analysis of a comprehensive collection of linked population health information including longitudinal, person-specific, de-identified data on BC’s 5 million residents. The data stewards prepared and authorised the release of the mortality data and helped to de-identify and aggregate the data from postal code to CT by using the Postal Code Conversion File (PCCF) (29). Age, sex, underlying cause of death (International Classification of Diseases, revisions 9 and 10), usual residence and location of death were extracted. Deaths were tabulated by 5-year age groups (0-4 years, 5-9 years, and 5-year bands up to 85+ years), sex, and cause of death for each CT. Underlying causes of deaths were mapped to the 21 causes in the second level of the Global Burden of Disease (GBD) Study cause hierarchy. All deaths that were initially assigned garbage codes in the GBD cause list (30) (31% of this dataset) were reclassified using a simple algorithm back into one of these 21 cause groups; the algorithm is based on linking the first 2-4 characters of the garbage codes with the first 2-4 characters of the ICD codes within the GBD level 2 groups through a hierarchal manner (e.g. first 4 character then 3 characters, etc.) This was appropriate given the intrinsic pattern already established within the ICD coding system and because the level 2 causes were broad enough for reclassification, unlike level 3 cause classifications (e.g. tuberculosis) or level 4 classifications (e.g. latent tuberculosis infection). A full list of the ICD codes for each cause can be found in the appendix.

Population counts by age group and sex for each CT in year 2016, 2011, 2006, 2001, 1996, and 1991 were collected from the Statistics Canada (31). We also used two covariates: proportion of population who identify as Aboriginal and the Material and Social Deprivation Indices (MSDI). The MSDI combines six different indicators chosen to reflect material deprivation, or the lack of everyday goods and commodities, and social deprivation, the fragility of an individual’s social network (32). All variables were collected from the Canadian Census for each census year since 1991 and interpolated between census years until 2016.

### Crosswalks and shapefiles

In 1991, there were 299 CTs in Metro Vancouver compared to 478 CTs in 2016. To derive estimates to a common set of boundaries, apportionment tables from Allen and Taylor (33) were used to generate death, population, and covariates estimates for the 2016 CT shapefile and applied to all previous census years in our study period (1991, 1996, 2001, 2006, 2011). This method uses population-based, areal, and dasymetric procedures to generate longitudinal sub neighbourhood-level population estimates. Certain CTs were removed or interpolated due to missing population data. To maximise the full data set, certain CT shapefiles and the corresponding data were merged to create stable units over time. See appendix for more details.

### Small area model

The model was adapted from a small area model applied in King County, Washington by Dwyer-Lindgren and colleagues (12) and fitted using Template Model Builder package (34) in R (v.3.6.1) (35). The small area model uses a Bayesian mixed-effect regression model to estimate all cause and cause-specific mortality. Multi-level Bayesian statistical procedures using both ‘fixed’ and ‘random’ parameters allow for small area life expectancy (LE) measures with smaller standard errors (22). This modeling approach of data pooling and spatial smoothing facilitates the ‘borrowing of strength’ between correlated geographic areas, age groups, and time periods to stabilise LE estimates. Age-specific mortality rates were normalised via direct standardization using the British Columbia 2016 Census population as a standard. The model specifications are:

$$\begin{array}{c} D_{j,t,a}∼Poisson\left(m_{j,t,a}\cdot P_{j,t,a}\right)\\ \log\left(m_{j,t,a}\right)=\beta_{0}+\beta_{1}\cdot X_{j,t}+\gamma_{1,a,t}+\gamma_{2,j}+\gamma_{3,j}\cdot t+\gamma_{4,j,t}+\gamma_{5,j}\cdot a+\gamma_{6,j,a} \end{array}$$

where *D_j,t,a_* is the number of deaths, *m_j,t,a_* the underlying mortality rate in CT *j*, year *t*, and age group *a*, and *P_j,t,a_* is the population; *β*_0_ is a fixed intercept; *X_j,t_* is the vector of covariates for CT *j* and year *t*; *β*_1_ is the associated vector of regression coefficients; *γ*_1,*a*,*t*_ are age-level and year-level random effects; *γ*_2,*j*_ are CT-level random effects; *γ*_3,*j*_ are CT-level random effects on year; *γ*_4,*j*,*t*_ are CT-level and year-level random effects; *γ*_5,*j*_ are CT random effects on age group; and *γ*_6,*j*,*a*_ are CT-level and age-level random effects. *γ*_1,*a*,*t*_, *γ*_2,*j*_, *γ*_3,*j*_, and *γ*_5,*j*_ were assigned conditional autoregressive priors to model the spatial phenomena of CTs and smooth the mortality rates by using information of adjacent CTs (36,37); and *γ*_4,*j*,*t*_ and *γ*_6,*j*,*a*_ were assigned mean-zero normal priors which allows for smoothing over age groups and time simultaneously (27,38). As described by Dwyer-Lindgren and colleagues (39), this model has been assessed using a validation framework and was shown to outperform other models based on bias, precision, and coverage of uncertainty intervals.

LE was calculated based on the estimated age-specific all-cause mortality rates using standard demographic techniques (40) to construct abridged life tables for each census tract and year. In addition, Horiuchi-Coale’s method (41) was used to extrapolate mortality rates for the open age group (42). Finally, we drew 1000 samples of the model parameters from the posterior distribution and used them to generate 1000 sets of mortality rates and LEs for each census tract, sex, and age group. From the 1000 age-standardised mortality rates and life expectancy values, we created point estimates from the median and 95% credibility intervals (CI) using the 2.5% and 97.5% values. To examine trends in inequality, we report life expectancy at birth and mortality rates at the 90^th^ percentile (P90 CTs) and 10^th^ percentile (P10 CTs). Using these measures, we also compute the absolute differences in inequality rates (P90-P10) and the relative inequality ratios (P90/10) for all 20 groups of mortality causes.

### Migration analyses

Without annual geospatial data linked to mortality data, most studies assume location of death is location of long-term residence. This study had the unique opportunity to link the mortality data with annual Consolidation File data from the Ministry of Health (43) through PopDataBC’s secure research environment (28) where information on location of residence is available annually as part of the mandatory health insurance program which covers nearly all residents. To assess the effects of migration on the LE estimates, two analyses were done by reassigning the location of residence based on where decedents lived in the past 5 years and 10 years prior to death. In contrast, the original LE estimates used the CT of residence last recorded in the vital statistics records. In the reassigned models, the CT where decedents resided most often within the study area in those two time periods (5 years and 10 years) was used as the CT of residence. In cases where a decedent had less than 5 years or 10 years of residential data (17.8% and 38.1% of the data, respectively), we reassigned based on the data that was available. The two reassigned models were subsequently compared to the original model to assess the effects of migration on the original LE estimates. A summary of all the datasets that were used with links to the sources has been detailed in Table 1.

## Results

After merging, there were 368 CTs in the final model. CT-level annual populations for Metro Vancouver varied from 40-19745 with a median value of 4315. Overall, we classified 350,094 of the death counts (99.8% of total death files) to the GBD ‘Level 2’ causes. Examples of unclassified deaths among the garbage codes that were not reclassified with our algorithm include blindness, cerebral palsy, and paraplegia. There were 0-167 deaths in each CT-year group, with a median of 22 deaths.

**Figure 1:**
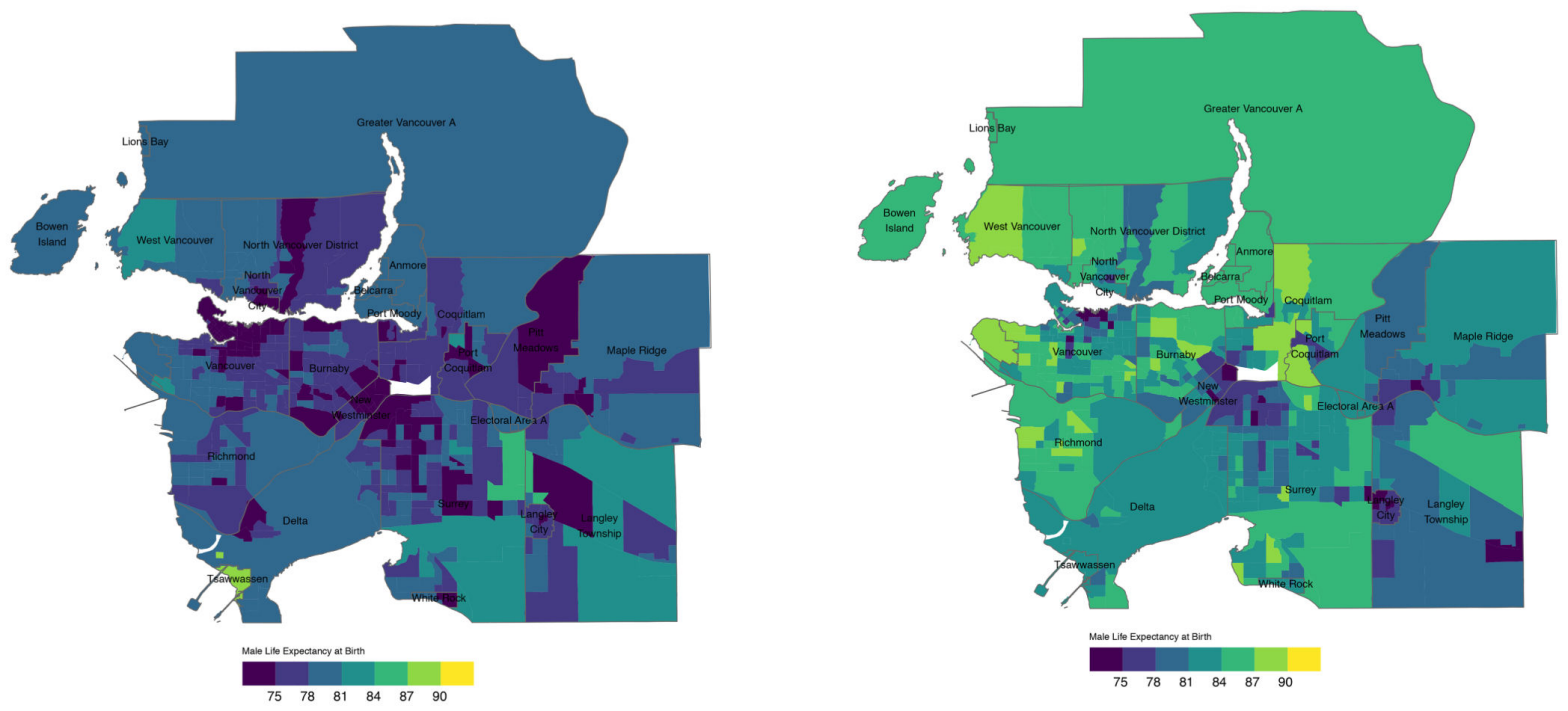
1991 and 2016 Male Life Expectancy at Birth (years)

In 2016, median male LE in Metro Vancouver was 82.5 (95% credibility interval: 80.2-85.6) years, and varied from 77.6 (77.1-78.1) years in the 10^th^ percentile (P10) to 87.1 (86.7-87.6) years in the 95^th^ percentile (P95). Male LE was mapped across Metro Vancouver for the years 1991 and 2016, which showed a decrease in the number of CTs with estimates of <75 years from 86 in 1991 to 11 in 2016 (Figure 1).

**Figure 2:**
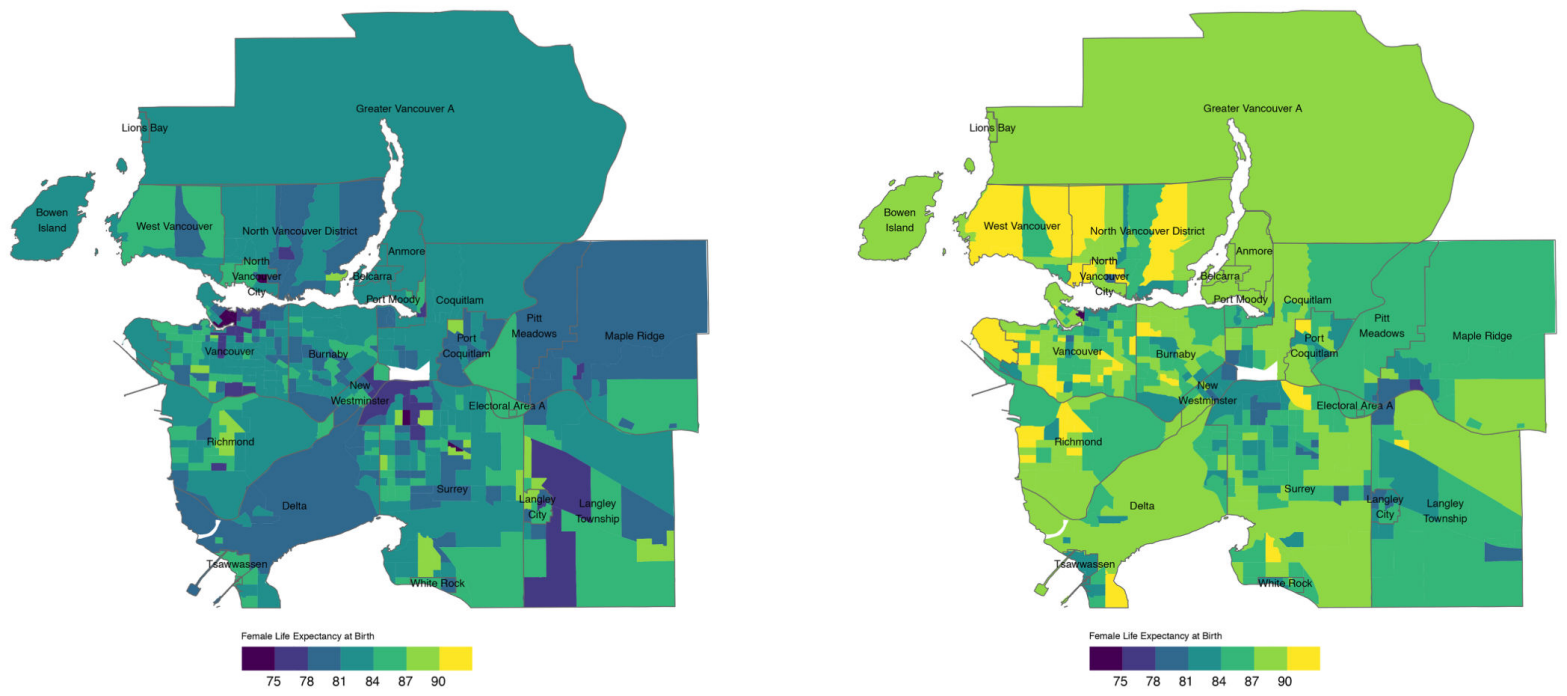
1991 and 2016 Female Life Expectancy at Birth (years)

For females, median LE in Metro Vancouver was 86.6 (84.0-89.8) years in 2016, and varied (P10-P90) from 82.5 (82.0-82.9) years to 90.8 (90.3-91.2) years. Female LE was mapped across Metro Vancouver for the years 1991 and 2016, and indicated a decrease from 6 in 1991 to 1 in 2016 in the number of CTs with LE estimates <75 years, while the number of CTs with estimates >90 increased from 1 in 1991 to 40 in 2016 (Figure 2).

**Figure 3:**
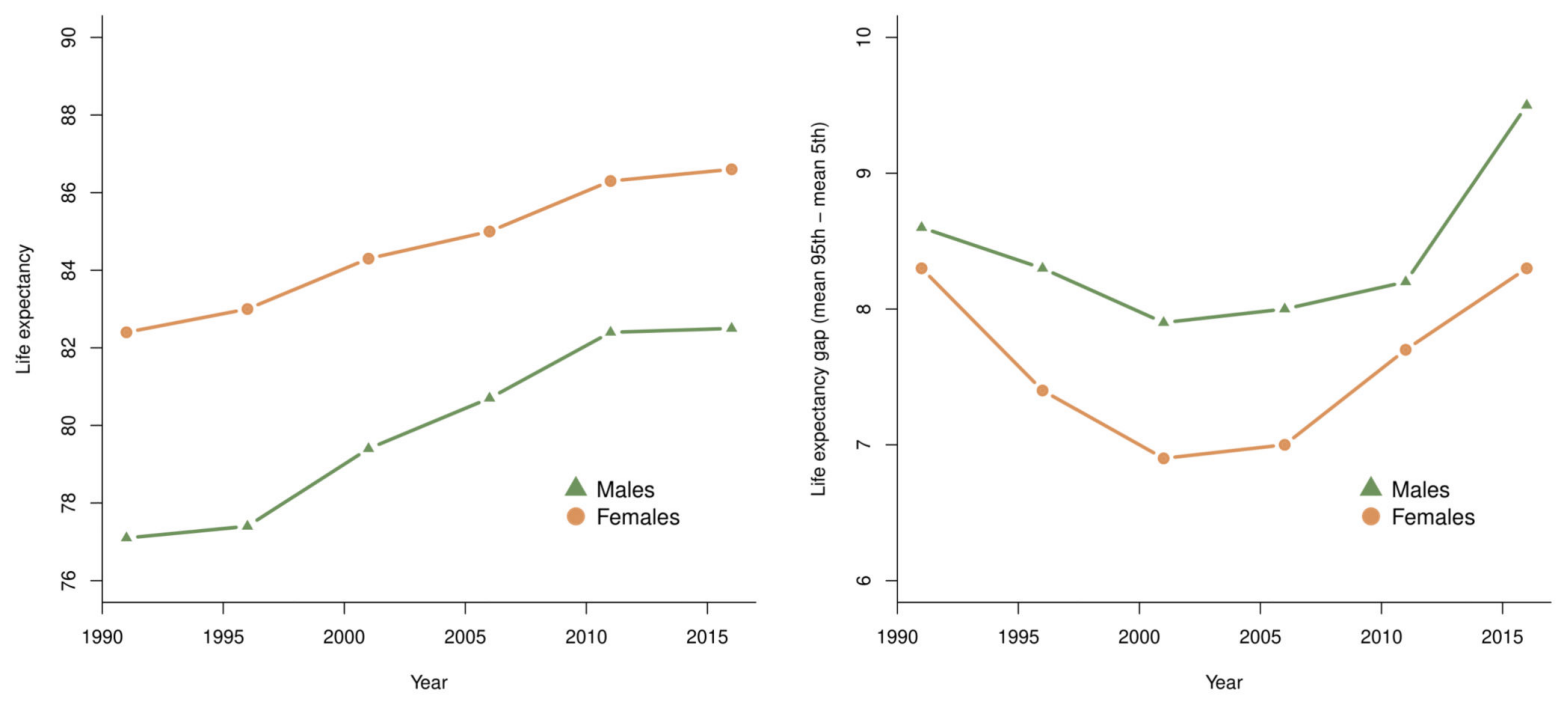
Historical trend of median life expectancy estimates (years) and inequality in life expectancy between the median 90^th^ and median 10^th^ CTs (years) for males and females

Between 1991 and 2016, there was a downward trend in the LE gap, whereby the lowest gap was observed in 2001 (6.9 years for females and 7.9 years for males), but this reversed and increased by 1.4-1.6 years between 2001-2016 (Figure 3). Over the entire period, the LE gap observed had increased for males by 0.9 years (9.5-year gap) and did not change for females (8.3-year gap). See appendix for more detailed LE results.

### Cause specific analysis

20 cause groups were used in the final analysis. Deaths from mental health disorders had extremely low counts and were excluded from the cause analyses.

**Figure 4:**
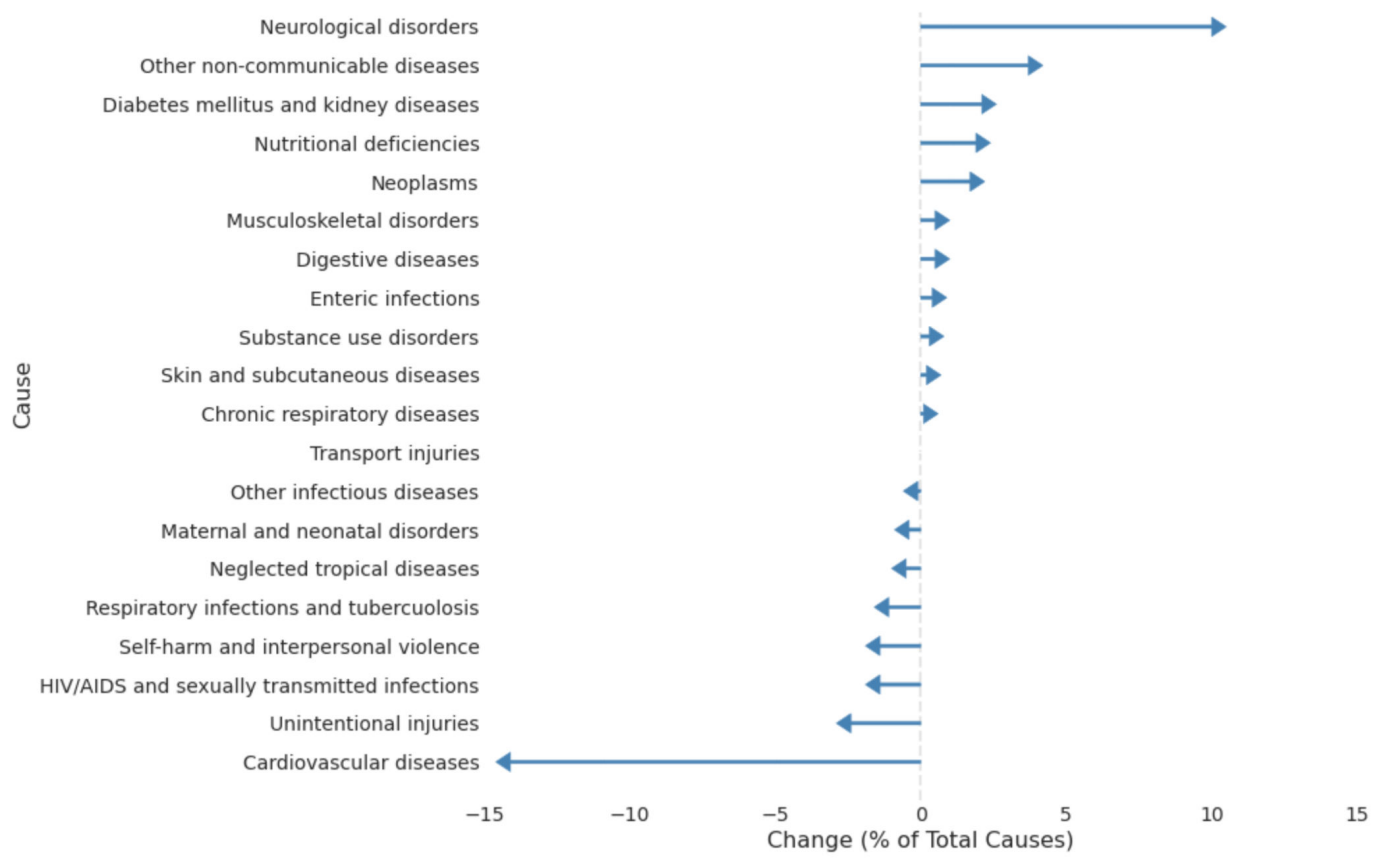
Change in Percentage of Total Causes from 1991-2016

**Figure 5:**
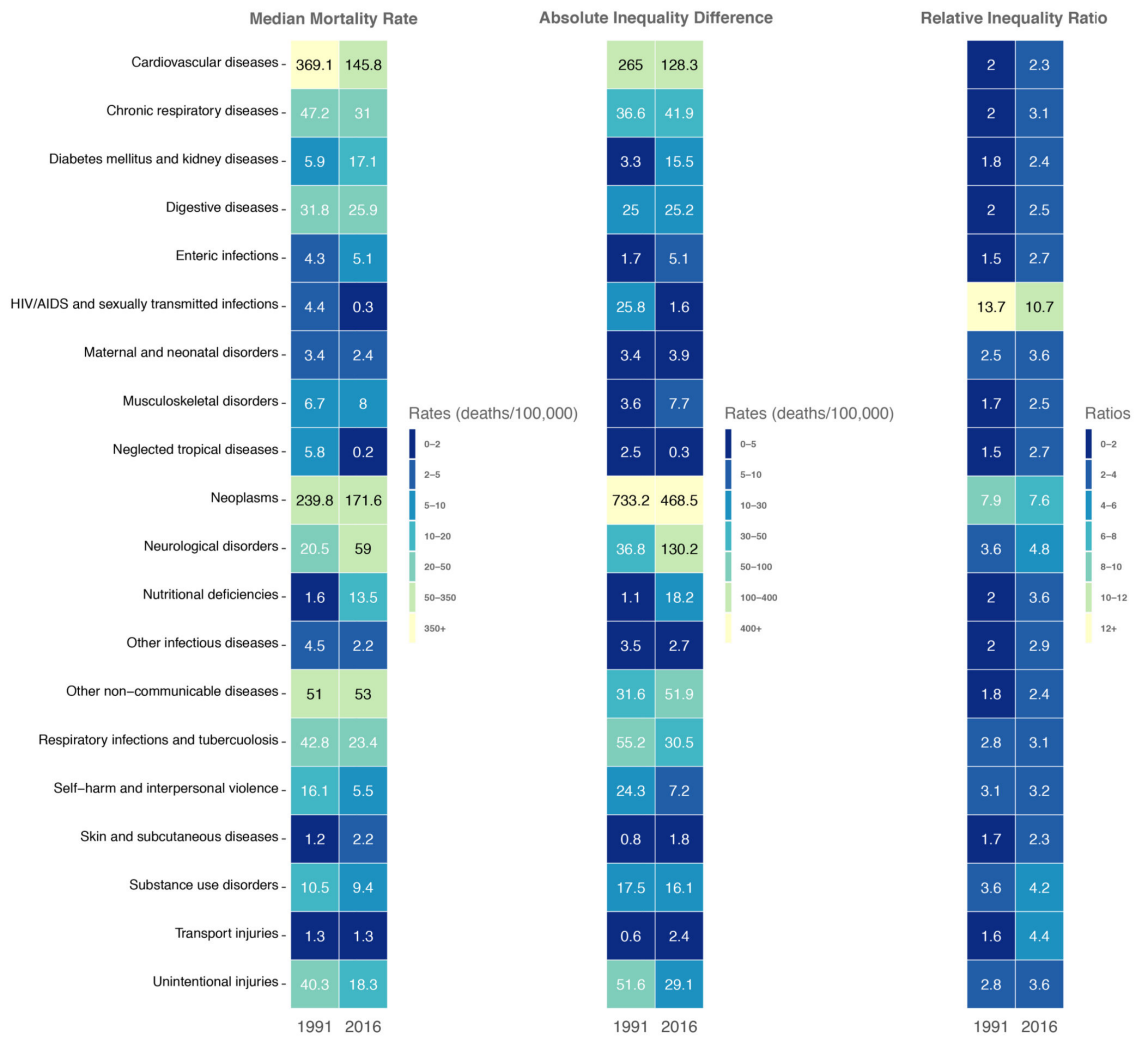
Inequalities for 20 causes of death in age-standardised mortality rate per 100,000 – Median, Absolute Inequality (90^th^-10^th^, deaths/100,000), and the Relative Inequality ratio (90^th^:10^th^)

In relation to percentage of total deaths, the two largest increases in causes of deaths from 1990-2016 were neurological diseases (10.0%) and other non-communicable diseases (3.7%) and the largest decreases were for cardiovascular diseases (-14.1%) and unintentional injuries (-2.4%) (Figure 4). Figure 5 summarises the results of the cause analysis by age standardised mortality rate per 100,000. Notably, neoplasms, cardiovascular diseases, and unintentional injury mortality rates decreased by 264.7, 136.7 and 24.7 from 1990 to 2016, respectively. Meanwhile, during the same period, the rates for neurological disorders, other non-communicable diseases, and nutritional deficiencies increased by 93.3, 20.3, and 14.1, respectively.

When comparing P90 and P10 rates in 1991 (Figure 5), three notable relative differences observed include HIV/AIDS and sexually transmitted infections at around 12.2 times higher for the P90 CTs (16.4, 95% CI: 13.2-20.7) compared to the P10 CTs (1.3, 1.0-1.8), maternal and neonatal disorders at around 7.1 times higher for the P90 CTs 7.1, 5.4-9.0) compared to the P10 CTs (1.0, 0.7-1.3), and neoplasms at around 5.6 times higher for the P90 CTs (600.2, 554.5-650.0) compared to the P10 CTs (131.3, 122.1-140.7).

The diseases driving inequality changed slightly by 2016; three relative differences observed were HIV/AIDS and sexually transmitted infections at around 17.4 times higher for the P90 CTs (1.2, 95% CI: 0.7-2.3) compared to the P10 CTs (0.1, 0.0-0.1), maternal and neonatal disorders at around 10.0 times higher for the P90 CTs (5.6, 4.1-7.7) compared to the P10 CTs (0.6, 0.4-0.8), and transport injuries at around 5.6 times higher for the P90 CTs (3.0, 1.9-4.8) compared to the P10 CTs (0.5, 0.3-0.9). By 2016, the absolute inequality increased the most for neurological (73.2), nutritional deficiencies (14.8), and other non-communicable diseases (12.7), and, while the relative inequality gaps increased for all diseases, except for neoplasms.

**Figure 6:**
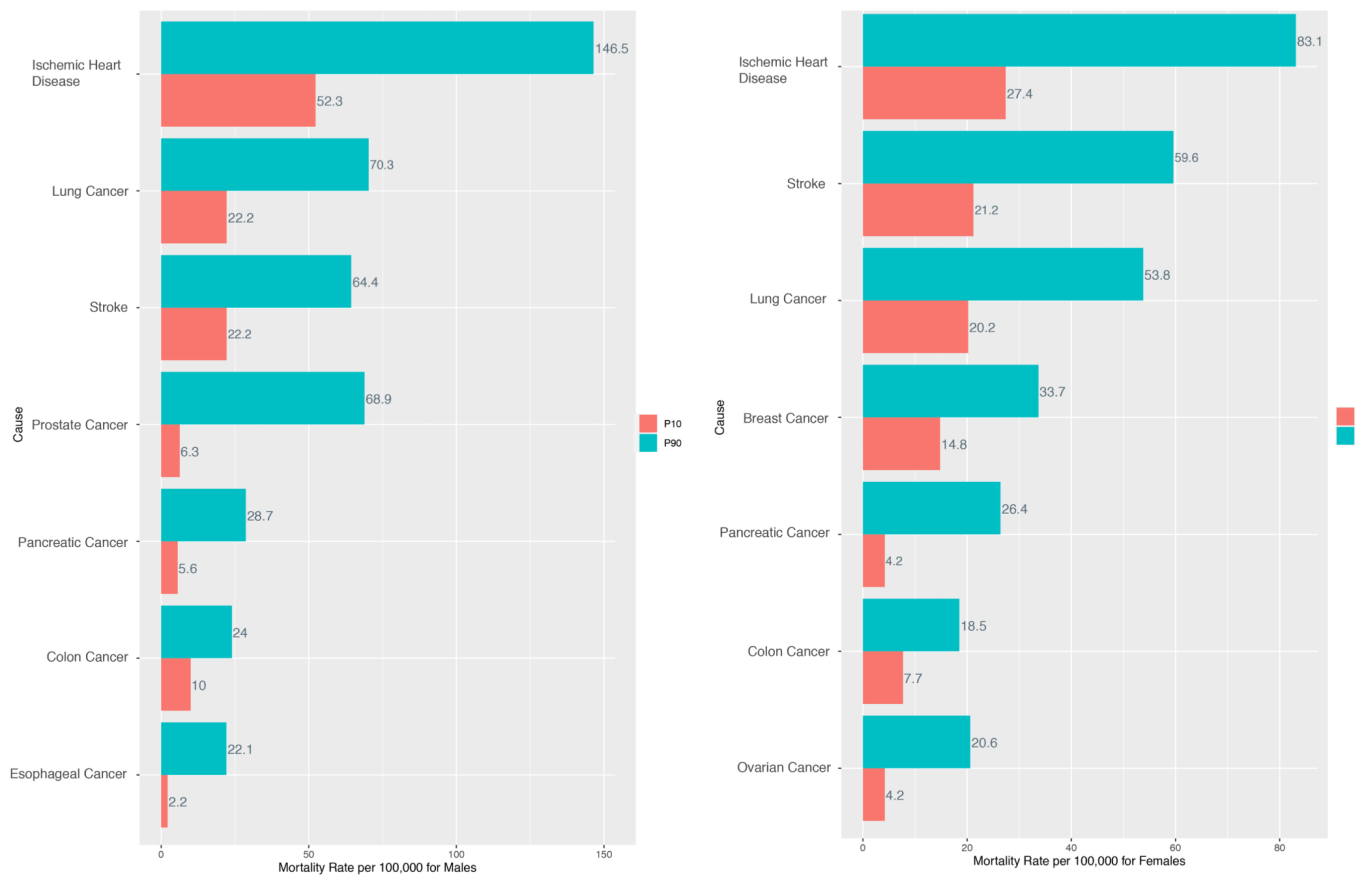
Inequalities for top 2 leading causes of cardiovascular deaths and top 5 leading causes of neoplasm deaths by sex – age-standardised mortality rate per 100,000 in 2016

Within cardiovascular diseases, the two leading causes of deaths were from ischemic heart disease (IHD) and stroke; mortality from IHD was 1.8 times higher for males (88.9, 95% CI: 51.7-154.5) compared to females (49.5, 28.3-84.6), but the relative difference was on average similar (2.9) (Figure 6). For stroke, absolute inequality (37.1) and relative inequality (2.9) were on average similar for both males and females.

From 1991 to 2016, mortality from pancreatic and lung cancer had some of the highest absolute inequality differences for both males (23.2-48.1) and females (22.2-30.8). Stark relative differences can be observed among males for deaths from prostate cancer, whereby CTs in P90 (68.8, 59.2-79.5) observed up to 10.9 times more deaths from prostate cancer compared to CTs in P10 (6.3, 5.3-7.3). Among females, the highest relative difference was observed for pancreatic cancer, whereby CTs in P90 (26.4, 23.0-30.3) observed 6.3 times higher deaths from pancreatic cancer compared to CTs in P10 (4.2, 3.6-4.8). See appendix for more details of the subset analyses.

**Figure 7:**
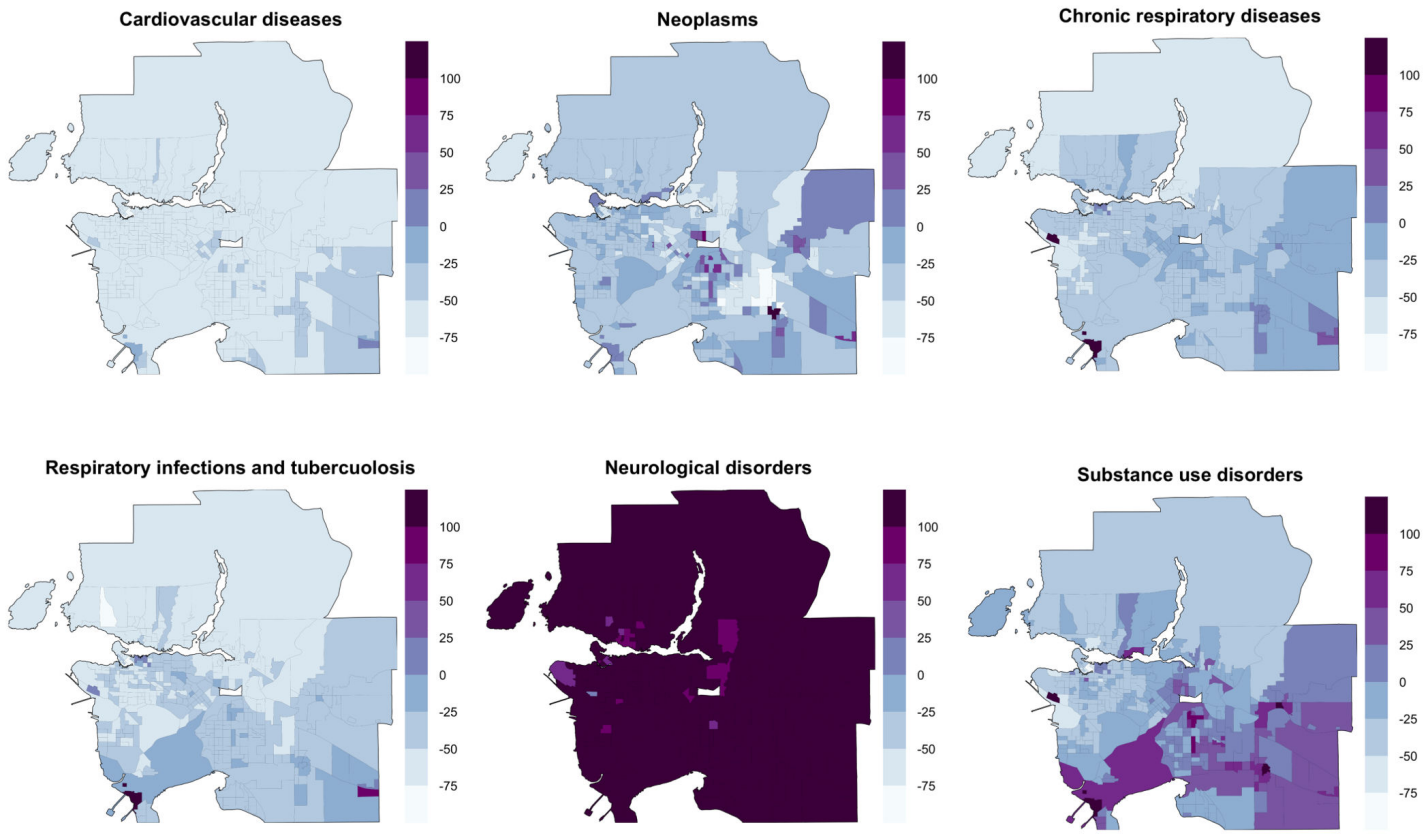
Spatial and temporal analysis of six different causes in Metro Vancouver – change in mortality rates from 1991-2016 (%)

Generally, rates for most causes decreased from 1991 to 2016 as population and the number of CTs increased (Figure 7). The notable exceptions include neoplasms, substance use disorders, and neurological diseases, where certain areas experienced more than 100% increase in mortality rates; the highest observed was more than four-folds increase for neurological diseases. The greater spatial differences in mortality changes may explain in part why deaths from certain diseases had higher inequality gaps by 2016 compared to their respective gaps in 1991 and to other causes. Certain suburban areas observed higher mortality rates for multiple causes, reflecting the population migration and commercial expansion of these neighbourhoods that were previously more rural.

### Migration Analyses

After reassigning the mortality dataset by duration of residence, whereby CT of residence of decedents were assigned based on the CT a decedent lived in the longest for 5 years and 10 years prior to death, we analysed the change and agreement of LE estimates in the reassignment model compared to the LE estimates in the original model (see appendix for plots). In the reassignment models, 14.5% of the CT sex-specific LE estimates changed by more than 5% when analysing a 5-year period and 20.3% changed by more than 5% using a 10-year period. Overall, we did not observe a substantial change in LE estimates even after reassigning residential address based on 10 years of residential data.

## Discussion

Within an urban area with one of the highest life expectancies (LEs) globally (26), we found substantial spatial and temporal variation in LE (up to 9.5 years for males and 8.3 years for females) and in numerous causes of death within Metro Vancouver. To our knowledge, this is the first study in Canada to comprehensively estimate mortality measures at the census tract (CT) level and simultaneously for multiple causes and years. Over this period, females not only lived longer than males, but the gap between CTs with some of the highest LE (90^th^ percentile, P90) and the lowest LEs (10^th^ percentile, P10) decreased during this period for females and increased for males, with inequality most recently at its highest for males over the 27-year study period. By cause, the observed historical trends show that by 2016, there were more increases among causes of death from aging and non-communicable diseases (NCDs) (e.g., neurological disorders, neoplasms, diabetes mellitus and kidney diseases) and substantial decreases were observed for infectious diseases (e.g., HIV/STD, neglected tropical diseases, and respiratory infections) along with deaths from unintentional causes. This is consistent with worldwide trends that show more than two-thirds of deaths are from NCDs, especially cardiovascular diseases, cancers, respiratory diseases, and diabetes (44). The WHO attributes this rise in NCD mortalities observed in our analysis and in other global cities around the world in part to rapid urbanisation, globalisation of unhealthy lifestyles, and population ageing.

Altogether, variation was still observed for all causes, the highest variation in absolute magnitudes of inequality were, as expected, for the top three causes of death: neoplasms, cardiovascular diseases, and neurological disorders, respectively. These NCDs that drive the absolute magnitude of inequality can further impede poverty reduction initiatives and exacerbate existing poverty by increasing direct household costs, including health care and lengthy treatments, and indirect costs, such as loss of income. Prostate and lung cancer contributed to the large absolute and relative inequality observed in neoplasm-related mortality, with the former contributing to up to eleven-folds higher mortality rate for men in P90 CTs compared to P10 CTs. Previous studies have shown that racial disparities may explain some of these variations; Black Americans compared to White Americans in the US were 50% less likely to receive treatment for high-risk prostate cancer (45) and people who identify as First Nations compared to non-Aboriginal adults were found to have more than two-folds higher excess mortality rate from cancers in Canada (46). In terms of relative inequality, we found large disparities for HIV/AIDS and sexually transmitted infections, maternal and neonatal disorders, and transport injuries, whereby some CTs in P90 experienced up to seventeen-folds, ten-folds, and five-folds, respectively, higher rates compared to CTs in P10. Although these causes had some of the lowest median mortality rates and contributed a decreased proportion of total deaths from these causes by 2016, these findings show that not all CTs experienced the same level of reduction of mortality over time. Some of these causes may also be driven by inequities, which allude to the systemic, unfair, and unjust differences in the distribution of health, social, and environmental resources, living conditions and opportunities in some CTs (47).

Within the center of the City of Vancouver, we observed a 10+ year gap in LE for males in CTs that are located within 5km of each other. Further, assessing these temporal trends is also important, as our analysis identified periods where LE inequality was decreasing (1991-2001) before increasing recently (2001-2016). Underlying societal trends, key changes to the health services and their delivery, urban policies, and/or economic tools enacted during these time periods may explain why inequality has increased since 2001 and particular for certain groups, such as males. For example, recent epidemiological research has documented the surge in unintentional poisonings, such as opioid drug overdose, which saw approximately 300% increase in deaths related to these causes from 2014-2016 and accounted for 32% of the decline in life expectancy for British Columbians (48). Another study in Quebec, Canada found similarly that lung cancer mortality increase inequality among women and mortality from HIV widened disparities among men (49), while a study in New Zealand found that cancers contribute to the sex difference in survival, especially colorectal screening among men (50). The results in this study quantified and visualised absolute and relative inequalities between P90 and P10 CTs to provide more understanding of the drug overdose epidemic in Vancouver, and other leading causes of inequalities, such as HIV/AIDS and sexually transmitted infections, maternal and neonatal diseases, and neoplasms.

These results on wide intra-urban gaps in Metro Vancouver, along with previously reported studies in the USA (12,39), UK (17,18), Netherlands (22), and Latin America (21), can be combined with spatially varying data on built environment, socioeconomic and municipal services to evaluate mechanisms underlying these observed inequalities whereby communities that are situated adjacent to one another may experience profound inequalities. For example, a previous study in England demonstrated that enacting an English health inequalities strategy was associated with a decline in geographic health inequalities (51). Urban planners can use these data to conduct additional analyses of the contributions of specific causes of death and their determinants to life expectancy changes and assess the appropriate interventions to address these inequalities. Previous studies found that access to methadone clinics decreased the odds of living in communities with higher opioid overdose rates (52), changes in patterns of land use that favoured gentrification lowered the use of HIV/STI testing and other sexual health services (53), and improved access to cancer screening, early diagnosis, and high quality treatment for all populations (54). The CT-level cause-mortality measures can be used to build on these previous studies by examining spatial correlates at finer spatial levels by cause of death and sex.

Assessing the interplay between disparities among racial/ethnic groups and disparities spatially may also yield valuable insights. In Canada, life expectancy was found to be lower among First Nations communities by an average of 16 years (55) compared to non-Aboriginal adults. In a cross-country comparison across Indigenous populations in New Zealand, Australia, Canada, and the United States, Canadian First Nation people experienced some of the highest mortality from intentional self-harm, pneumonia, and influenza (56). Several US-based studies also observed that people of colour, including non-Hispanic American Indians and Alaskan Natives, Black individuals and Asians and Pacific Islanders, experienced large increases over time in mortality rates from more than 10 causes, with notable larger contributions from fatal drug overdoses, alcoholic liver diseases, suicides, and hypertensive diseases compared to other causes (57–59). Vancouver, like many other cities around the world, is racially/ethnically segregated and has ‘minority group enclaves’ (60), and therefore racial/ethnic differences in mortality likely explain in part the observed spatial variations. A systematic review in Canada found ethnic differences in cardiovascular disease risk factors; for example, compared to white individuals, there was a greater prevalence of hypertension among black and Filipino individuals and a greater prevalence of diabetes in Hispanic and Filipino individuals (61). Another study also found ethnic differences in survival for female cancers, which reported higher survival rates for colorectal cancer among South Asian women and higher survival rates for breast and cervical cancer for Chinese women compared to all BC women (62). Future studies should disaggregate these small area mortality estimates by multiple ethnicities, ages, and causes to further examine social and cultural factors that have differentially impacted racialized communities in the past, such as during the COVID-19 pandemic in Canada (63).

### Limitations

As with most administrative data, the death counts, and covariates used are subject to misreporting and should be treated as estimates. This should also be factored in for the intercensal interpolations and crosswalk files that were used. Essentially, by using crosswalk data, we created estimates of populations and deaths in areas that may not have existed in historical years, especially 1991-1996. As we endeavored to retain as much of the 2016 CT shapefile as possible, this may have affected the historical estimates CTs outside the urban core more than the most recent year of data, especially 2016. Deaths occurring outside of British Columbia were not included, although this is likely not a substantial factor. Similarly, the migration analysis only accounts for migration within the study area where data was available.

As in any area-based analysis, there is likely variation among households within the same CT. Further, the design of this study does not infer causality – for example, whether individuals or newcomers with similar characteristics and health behaviours are choosing or forced to live in the same communities, or if the health of the community residents are driven by the features of the surrounding environments, such as housing cost, access and quality; green space; or access to health promoting facilities. Moreover, the life expectancy measures do not account for quality of life.

For cause-specific mortality trends, coding and diagnostic practices have also changed over time. Notably, there are heterogeneity in coding accuracy and need for more validation of case definitions for neurological conditions (64). Different algorithms have been used to address ‘garbage codes’ or deaths that one cannot technically die from. In this study, we were unable to apply the exact methods that have been used in similar studies in the United States (US) (20) due to context-specific literature, expert opinion, ICD rules, and knowledge about the disease used to inform prior statistical models and algorithms. Around 33% of the deaths were initially classified to garbage codes in this study but were reclassified to GBD Level 2 causes using a simple reclassification algorithm based on the first 2-4 characters of the ICD codes in the existing GBD level 2 cause groups. The GBD level 2 are aggregate groupings of disease and injury (e.g. cardiovascular disease), whereas the GBD level 3 categorisation are more specific causes (e.g. stroke). The assumption implicit is that the death likely falls in the same broad-level cause category (e.g. respiratory infections), which could not have been done if we used a more detailed or specific category, such as GBD level 3 causes (e.g. drug-susceptible tuberculosis). Given that there is no gold standard in Canada or the US for comparisons, it was our decision to reclassify the deaths back into the same category. Nevertheless, there is still possible misclassification bias of garbage codes that span across multiple level 2 causes (e.g. injuries where intent is undetermined or sepsis). Future studies with access to medical records or autopsy reports could validate these findings.

## Conclusion

This study was undertaken in response to calls (65) for more research and solutions to tackle historical and present health disparities. These disparities may result from systemic injustices, such as inequitable health care and nutritional food access, and social and environmental determinants, such as income and race inequality and urbanisation. These factors affect not only mortality rates of chronic diseases over time, but they were also drivers during recent acute health crises, such as the opioid overdose and covid-19 pandemic. By quantifying neighbourhood- and sub-neighbourhood-level indicators of health, we observed spatial heterogeneity and clusters of cause-specific deaths in one of the healthiest cities in one of the healthiest countries of the world. Future research can use these mortality measures to explain drivers of past health disparities, potentially predict and intervene against future disparities, and to identify susceptibility to future acute events such as another infectious disease outbreak or climate-induced shock. In doing so, we can implement appropriate regulatory and fiscal solutions to ensure *health equity is in all policies* and build capacity at the level that arguably matters the most – within households and communities.

## Online dissemination tool

An RShiny visulisation tool is available for dissemination of study results.

## Supplementary Material

Supplementary Material

## Figures and Tables

**Table 1:** Summary of the datasets used with details on years, variables, and source

Dataset	Years	Variables	Source
BC Vital Events and Statistic: Deaths	1990-2016	Age, sex, underlying cause of death, year of death, census tract of death, census tract of residence, study ID	BC Ministry of Health via PopDataBC
BC Consolidation File	1986-2016	Study ID, census tract of residence	BC Ministry of Health via PopDataBC
Canadian Census	1991, 1996, 2001, 2006, 2011, 2016	Population, age, sex, year, census tract	Statistics Canada
Material and Social Deprivation Index	1991, 1996, 2001, 2006, 2011, 2016	Material deprivation index score, social deprivation index score, census tract	Canadian Urban Environmental Health Research Consortium (CANUE)
