# Supplementary material for: A spatiotemporal analysis of inequalities in life expectancy and 20 causes of mortality in sub-neighbourhoods of Metro Vancouver, British Columbia, Canada, 1990-2016
Source: Health Place. Author manuscript; Available in PMC 2023 Sep 22. (PMC7615115; doi:10.1016/j.healthplace.2021.102692)
Supplement: Supplementary Material [file EMS187698-supplement-Supplementary_Material.pdf]

Supplementary Material

Missing Population in the following Census Tracts

Census tracts	Years	Action
58	2006-2011	Linear interpolation
161.07	2011-2016	Linear interpolation
251.01	2011-2016	Linear interpolation
290.06	2011-2016	Linear interpolation
400.07	2011-2016	Linear interpolation
400.08	2011-2016	Linear interpolation
270	1990-2016	Excluded

Merging CTs criteria:

The first criteria that was used was to merge CTs that had less than 150 people or death counts greater than 0 and less than 1 per CT-age group. Of all contiguous CTs, the selected CTs were merged with a neighbouring CT that had the smallest death or population count to also ‘strengthen’ the neighbouring CTs. The second criteria were to merge CTs with the low death rates (5th percentile) in the open age group (85%), which have been shown to affect standard demographic techniques in populations that are living longer (Scherbov, 2011). For this criterion, the CTs were merged with contiguous CTs with the highest mean mortality rate in the 85+ category to strengthen the selected CTs. This decision was made as merging with a contiguous CT with the smallest mortality rate in the 85+ age group did not strengthen the CTs enough to change the results.

Scherbov, S. and Ediev, D., 2011. Significance of life table estimates for small populations: Simulation-based study of standard errors. Demographic Research, 24, pp.527-550.

All causes as a proportion of total deaths in the dataset

Causes	Percentage of total deaths (1990)	Percentage of total deaths (2016)	Change (1990-2016)
Neoplasm	26.8	28.5	1.7
Cardiovascular	38.3	24.1	-14.1
Neurological	2.3	12.4	10.0
Other non-communicable diseases	5.2	8.9	3.7
Respiratory	5.0	5.1	0.1
Digestive	3.6	4.1	0.5
Respiratory Infection	5.1	4.0	-1.1
Unintentional	5.4	3.0	-2.4
Diabetes mellitus	0.5	2.6	2.1
Nutrition	0.2	2.1	1.9
Substance Use	1.3	1.5	0.3
Musculoskeletal	0.6	1.2	0.5
Intentional	2.1	0.8	-1.4
Enteric	0.3	0.7	0.4
Infectious	0.4	0.3	-0.1
Skin	0.1	0.3	0.2
Maternal / Neonatal	0.7	0.2	-0.4
Injury / Transport	0.1	0.2	0.0
HIV / STD	1.5	0.1	-1.4
Neglected Tropical Diseases	0.5	0.0	-0.5

Median life expectancy at birth for males, females, and both; 90th percentile (P90) and 10th percentile (P10) of Census Tract median life expectancy estimates and 95% uncertainty intervals (1991-2016); Gap (P90-P10)

Year	Males	P90	P10	Gap	Females	P90	P10	Gap
1991	77.1 (74.5-79.9)	80.9 (80.5-81.4)	72.3 (71.8-72.8)	**8.6**	82.4 (79.7-85.5)	86.5 (86.0-87.1)	78.3 (77.8-78.8)	**8.3**
1996	77.4 (75.1-79.9)	80.9 (80.5-81.3)	72.6 (72.1-73.1)	**8.3**	83.0 (80.5-85.7)	86.3 (85.9-86.8)	78.9 (78.5-79.4)	**7.4**
2001	79.4 (77.3-81.9)	82.9 (82.5-83.3)	75.0 (74.6-75.5)	**7.9**	84.3 (81.9-86.8)	87.4 (87.0-87.8)	80.4 (80.0-80.8)	**6.9**
2006	80.7 (78.6-83.0)	84.3 (83.9-84.7)	76.3 (75.8-76.7)	**8.0**	85.0 (82.7-87.5)	88.2 (87.8-88.6)	81.2 (80.8-81.6)	**7.0**
2011	82.4 (80.3-84.9)	86.2 (85.8-86.6)	77.9 (77.5-78.4)	**8.2**	86.3 (84.0-89.2)	90.0 (89.6-90.5)	82.4 (82.0-82.8)	**7.7**
2016	82.5 (80.2-85.6)	87.1 (86.7-87.6)	77.6 (77.1-78.1)	**9.5**	86.6 (84.0-90.0)	90.8 (90.3-91.2)	82.5 (82.0-82.9)	**8.3**

Inequalities for 20 causes of death in age-standardised mortality rate per 100,000 – Median, Absolute Inequality (90th-10th), and Relative Inequality ($90^{th}/10^{th}$)

	Age standardised mortality rate per 100,000 population						
Cause of death	Median (1991)	Median (2016)	Change (1991-2016)	1991 Gap (90th-10th) - absolute	1991 Gap ($90^{th}/10^{th}$) - ratio	2016 Gap (90th-10th) - absolute	2016 Gap ($90^{th}/10^{th}$) - ratio
Cardiovascular diseases	369.1	99	-223.3	226.1	1.8	116.6	2.2
Chronic respiratory diseases	47.2	19.6	-16.3	35.1	2	34	2.7
Diabetes mellitus and kidney diseases	5.9	11	11.2	3.6	1.9	14.4	2.3
Digestive diseases	31.8	17	-5.8	22.3	2	22.9	2.3
Enteric infections	4.3	2.9	0.7	2.2	1.7	5	2.7
HIV/AIDS and sexually transmitted infections	4.4	0.1	-4.1	15	12.2	1.1	17.4
Maternal and neonatal disorders	3.4	0.6	-1	6.1	7.1	5.1	10
Musculoskeletal disorders	6.7	4.7	1.4	4.7	2	8.3	2.8
Neglected tropical diseases	5.8	0	-5.6	5	2.4	0.3	7.6
Neoplasms	239.8	95.9	-68.2	469	4.6	271.8	3.8
Neurological disorders	20.5	33.3	38.5	24.8	3.6	97.9	3.9
Nutritional deficiencies	1.6	7.2	11.8	1.2	2.2	16	3.2
Other infectious diseases	4.5	1.1	-2.3	4.2	2.5	2.9	3.7
Other non-communicable diseases	51	35.4	2	33.9	1.9	46.7	2.3
Respiratory infections and tubercuolosis	42.8	13.9	-19.4	47.2	2.7	27.1	3
Self-harm and interpersonal violence	16.1	3	-10.6	1.2	2.5	6.6	3.2
Skin and subcutaneous diseases	1.2	1	0.9	0.8	2.6	2.8	3.8
Substance use disorders	10.5	4.3	-1.2	14.4	3.5	15.2	4.6
Transport injuries	1.3	0.5	0.1	1	2.4	2.5	5.6

Unintentional injuries	40.3	10.7	-21.9	37.1	2.4	23.8	3.2

Inequalities for 2 causes of cardiovascular deaths and 7 causes of neoplasm deaths in age-standardised mortality rate per 100,000 in 2016

	Age standardised mortality rate per 100,000 population (2016)					
**Subset cause of neoplasm deaths**	**Median (Females)**	**Gap (90th-10th) - absolute**	**Gap ($90^{th}/10^{th}$) - ratio**	**Median (Males)**	**Gap (90th-10th) - absolute**	**Gap ($90^{th}/10th$) - ratio**
**Neoplasm**						
Lung	**33.4**	30.8	2.7	**41.2**	48.1	3.2
Prostate	--	--	--	21.5	62.5	**10.9**
Breast	22.8	18.9	2.3	--	--	--
Colon	12.2	10.9	2.4	15.7	13.9	2.4
Pancreas	11.2	22.2	**6.3**	13.6	23.2	5.2
Esophagus	--	--	**--**	7.8	19.9	9.9
Ovary	10.2	16.5	5.0	--	--	--
**Cardiovascular**						
Ischemic heart disease	49.5	51.7	3.0	88.9	94.2	2.8
Stroke	35.6	35.2	2.8	39.0	42.2	2.9

Table of ICD 9 and 10 Codes for 20 Cause Groups and 9 Subset Causes

Adapted from GBD Level 2 Causes – garbage codes were reclassified back to the level 2 causes.

CAUSES	ICD9	ICD10
**Cardiovascular**	410, 4149, 4415, 4140, 4341, 4413, 4349, 4148, 4249, 4254, 431, 4255, 4029, 4409, 413, 4275, 4439, 4299, 4241, 4410, 4293, 4290, 4240, 4292, 4259, 4476, 4273, 4370, 4340, 430, 4329, 4331, 411, 4371, 4411, 4242, 4279, 4379, 4298, 4414, 4479, 435, 3949, 4291, 3940, 4402, 3979, 4416, 396, 4274, 3912, 4229, 3970, 4592, 4230, 4372, 4431, 4519, 412, 3989, 4532, 4512, 4141, 4511, 4578, 4253, 4321, 4271, 4472, 4429, 4239, 4210, 4373, 4599, 4278, 4232, 4549, 4270, 4330, 4251, 4400, 4590, 4538, 4518, 3941, 452, 3950, 4422, 4209, 4428, 4478, 4412, 4540, 4530, 4489, 4378, 3942, 4471, 4539, 4231, 4423, 3910, 4531, 4421, 4338, 4296, 3951, 4533, 4300, 4408, 4219, 4401, 4294, 4021, 4571, 4302, 4020, 4375, 4301, 3952	I219, I64, I48, I698, I249, I248, I259, I251, I509, I694, I429, I500, I713, I519, I209, I718, I639, I250, I516, I709, I38, I711, I469, I258, I619, I714, I672, I609, I442, I629, I611, I252, I739, I693, I119, I340, I802, I710, I255, I702, I110, I350, G459, I499, I613, I719, I607, I740, I712, I059, I745, I514, I635, I600, I080, I776, I099, I420, I359, I319, I200, I091, I358, I691, I517, I633, I518, I351, I341, I472, I638, I422, I620, I212, I311, I779, I330, I634, I459, I426, I460, I749, I424, I401, I723, I615, I071, I632, I606, I495, I830, I461, I728, I208, I820, I671, I400, I501, I050, I614, I318, I700, I809, I99, I743, I729, I872, I253, I089, I083, I608, I081, I409, I839, I741, I692, I716, I771, I724, I772, I309, I421, I428, I878, I673, I803, I715, I618, I214, I81, I058, I778, I690, I828, I864, I675, I060, I490, I079, I051, I078, I018, I352, I738, I456, I801, I731, I471, I701, I313, I440, I281, I612, B332, I610, I781, I621, I515, I254, I201, I213, I348, I602, I510, I498, I630, I210, I829, I458, I443, I229, I308, I722, I361, I748, I788, I256, I052, I425, I676, I631, I310, I730, I451, I770, I082, I513, G458, I871, I312, I832, I744, I069, I831, I368, I636, I211, I720, I511, I301, I288, I822, I241, I088, I601, I489, I470, I447, I349, I482, I481, I480, I603, I371, I441, I454, I5000, I423, I4209, I605

**Diabetes mellitus**	4049, 4039, 5839, 7530, 585, 5819, 5829, 7531, 7533, 4040, 5838, 5899, 5832, 5809, 5828, 5831, 5813	N19, E119, N179, I120, I132, N189, I10, N429, E109, N883, E105, N188, E102, E101, N059, N180, Q613, I131, E115, Q612, E113, E112, E104, N824, E103, I139, N136, N739, E107, N135, N051, N171, N132, N139, N62, E117, N433, E116, E111, N133, N170, E114, N138, I129, N421, E106, N719, Q614, N828, N359, N058, N019, N134, Q619, Q618, N482, E100, N057, N137, N053, N055, N052, N185, N184, N823, N735, N508, I130, E108, E118, N001, N731, N048

**Digestive**	5733, 5314, 5609, 5711, 5520, 5319, 5579, 5713, 5770, 5728, 5621, 5715, 5325, 5712, 5350, 5602, 5742, 5339, 5570, 558, 5400, 5324, 5608, 5710, 5714, 5303, 5368, 5335, 5724, 5523, 5751, 5301, 5739, 5779, 5509, 5326, 5355, 5315, 556, 5722, 5761, 5533, 5353, 5745, 5718, 5334, 5771, 4560, 5528, 5329, 5716, 5762, 5501, 5603, 566, 5719, 5571, 5308, 5741, 5529, 5744, 5305, 5321, 5641, 5759, 5750, 5309, 5351, 5693, 5304, 5601, 5768, 5302, 5316, 5300, 5370, 5559, 5500, 5694, 5307, 5648, 5647, 5550, 5754, 5306, 5692, 5354, 4561, 5521, 5330, 5379, 5369, 5723, 5695, 5640, 5336, 5729, 5799, 5738, 5691, 5740, 5720, 5401, 5790, 5778, 5764, 5332, 5697, 5539, 5519, 5758, 5730, 5772, 5344, 5651, 5532, 4556, 5620, 5734, 5378, 5409, 5769, 5600, 5649, 5791	K922, K254, K265, K746, K703, K529, K509, K264, K709, K559, K810, K704, K829, K297, K769, K804, K413, K625, K566, K552, K550, K830, K274, K20, K275, K701, K219, K831, K85, K565, K801, K409, K223, K802, K560, K909, K210, K403, K579, K519, K220, K259, K578, K429, K319, K902, K743, K767, K224, B181, K296, K350, K860, K819, K562, K318, K449, I850, K255, K267, K928, K868, B182, K564, K226, K440, K279, K461, K803, K37, K768, K567, K460, K521, K623, K805, K766, K269, K593, K30, K276, K572, K292, K628, K222, K563, K221, K469, K759, I848, K311, K863, K225, K228, K745, K828, K551, K861, K573, K629, K920, K089, K660, K290, I859, I849, K590, K266, K316, K929, K822, K668, K501, M911, K900, K256, K711, K740, K613, K420, K291, K838, K754, K661, K833, K561, K229, K359, K869, K610, K611, K270, K310, K626, K839, K421, K811, K051, K852, K859, K700, K250, K088, K294, K029, K823, K284, K570, K599, K571, K414, K862, K800, K518, K298, K260, K295, K761, K039, K719, K227, K901, K589, K315, K921, K441, K624, K605, K358, K261, K056, K450, K389, K352, K758, K510, K851, K718, K353, K317, K36, K669, K598, K289, K643, K458, K604, K280, K528
**Enteric**	4589, 4580	A047, A498, A419, A048, A491, A410, R13, A499, A310, A415, A414, A418, A412, R190, A490, A09, R198, A430, R100, R104, A044, A045, A073, A319, A099, A042, A438, A413, A439, A411, A049
**HIV/STD**	436, 4449, 4280, 438, 4281, 0421, 0429, 0420, 4289, 0422, 0449, 4441,	B203, B201, B207, B206, B227, B200, B238, B205, B212, B202,

	4442, 4440, 4260, 0433, 4269, 0970, 0431, 4267, 541, 4284, 4266, 0440, 4261, 4268	B232, B208, B219, B213, B222, B218, B217, A539, B020, B209, A509, B211
**Infectious**	5679, 4151, 0408, 0362, 0709, 797, 0469, 4019, 725, 0703, 7226, 3229, 0785, 052, 3202, 0705, 0461, 4150, 7240, 0410, 0549, 5672, 3239, 7289, 7219, 1363, 7211, 0499, 1369, 0539, 0360, 7288, 3209, 0531, 3201, 3659, 0704, 5680, 0702, 0462, 0701, 7291, 7200, 0790, 3208, 5280, 0479, 0270, 5688, 0706, 5671, 0543, 3753, 7210, 5272, 0742, 4010, 5225, 1390, 7711, 0463, 1398, 3760, 7281, 5231, 5289, 7229, 3690	B942, K729, B029, B449, B49, G049, A810, G588, K732, B159, K738, B349, A394, B24, B448, B169, B171, B004, B210, K720, A084, B59, K739, G048, B162, B948, K760, K750, B377, B250, G450, B91, G218, G001, B371, B441, B457, G002, A879, B941, B451, G008, K753, G528, G529, B341, B479, A081, B348, G009, B220, B582, B199, B450, A86, A321, G589, B027, B003, A390, B338, B258, K721, B019, K763, G219, A858, B465, B378, G587, B270, B370, A812, B259, B180, B447, B279, B009, B011, G210, G003, B374, G439, B379, B382, G211, B022, G214, B023, G14, B440, P353, B376, G08, A327, B375, B333, B589, A021, A083, B369, P372
**Injury/Transport**	E8052, E8415, E8413, E8320, E8426, E8321, E8447, E8311, E8333, E8329, E8388, E8068, E8361, E8309, E8437, E8300, E8301, E8302, E8417, E8389, E8405, E8419, E8322, E8310, E8412, E8048, E847, E830, E8312, E8059, E848, E8422, E8319, E8442, E8380, E8381	V092, V899, V892, V099, V685, V494, V445, V031, V929, V877, V499, V909, V865, V294, V905, V485, V435, V486, V479, V920, V436, V958, V595, V284, V279, V050, V239, V878, V913, V594, V041, V296, V495, V961, V475, V476, V234, V183, V746, V194, V959, V446, V254, V586, V968, V869, V535, V224, V849, V051, V198, V585, V786, V290, V274, V840, V536, V299, V285, V439, V599, V852, V575, V545, V030, V114, V235, V180, V484, V244, V694, V576, V396, V144, V845, V020, V456, V675, V489, V546, V874, V952, V405, V134, V950, V184, V275, V449, V903, V059, V139, V953, V902, V949, V040, V670, V635, V890, V860, V021, V587, V149, V471, V870, V861, V091, V686, V553, V104, V090,

		V906, V204, V972, V919, V960, V589, V925, V800, V245, V498, V131, V785, V154, V645, V443, V174, V525, V011, V835, V441, V684, V922, V887, V482, V477, V837, V093, V809
**Intentional**	E9520, E9554, E9571, E9689, E9530, E954, E9654, E9503, E9504, E9580, E966, E956, E963, E9589, E9570, E9505, E9500, E9552, E9501, E9509, E9521, E9651, E9507, E970, E9583, E9688, E9502, E9551, E9682, E9581, E9588, E9531, E9600, E9550, E975, E9680, E9538, E9585, E9681, E9679, E9559, E9579, E9650, E9584, E9506, E9582, E969, E964, E9539, E9601, E9511, E9671, E9572, E959, E9670	Y86, X74, X70, Y899, Y09, X80, X60, X71, Y850, Y33, Y12, Y20, Y871, X78, X72, X73, Y30, Y11, Y00, Y32, Y08, X76, Y350, Y04, X75, Y26, Y870, Y14, Y16, Y21, Y31, Y24, Y34, Y872, Y17, X79, Y28, Y03
**Maternal/Neonatal**	769, 7615, 7650, 7775, 7649, 7612, 7621, 7627, 7686, 7798, 7651, 7708, 7707, 7704, 7600, 7689, 7624, 7638, 7780, 7728, 7670, 7610, 7703, 7611, 7718, 7761, 7626, 7620, 7684, 7701, 7623, 7799, 7602, 7762, 7628, 7685, 7744, 7717, 7721, 6731, 7765, 7608, 6467, 7702, 7789, 6713, 6412, 7640, 7776, 7710, 7778, 7636, 7618, 6486, 7633, 7700, 7709, 7680, 7622	P027, P220, P968, P240, P073, P011, P369, P368, P291, P021, P010, P209, P524, P523, P548, P022, O754, P251, P072, P219, P038, P529, P269, P050, P298, P279, P271, P278, P280, P559, P910, P90, P015, P964, P832, P508, P024, P059, P912, P77, P250, P035, O751, P290, P012, P229, P018, P000, O998, P039, P788, P528, O881, P399, P293, P070, P916, P130, P020, P360, O660, O429, P031, P026, O97, O009, P613, P112, O994, P809, P001, P504, P569, P361, P522, P003, P789, P002, P365, P008, P159, P918, P288, P614, P599, O721, O141, P201
**Mental**	3075, 3071	F508, F500, F509, F54, F502
**Musculoskeletal**	4160, 7100, 7148, 4169, 7101, 7140, 7339, 4462, 7143, 7330, 4460, 7109, 6954, 7104, 7331, 4464, 4465, 4374, 7102, 4466, 4467, 4161, 7110, 4461, 7301, 7141, 7103, 7332, 7108	I269, M069, M199, I679, M419, I279, M819, I270, M321, I271, M809, I678, I670, M349, M348, M954, M628, M869, M479, M329, I677, M359, M313, M513, M844, M009, M139, M431, M310, M462, M316, M889, M808, M353, M311, M259, M159, M341, M052, M192,

		M300, M600, M45, M866, L930, M478, M169, M625, M469, M130, M480, M189, M549, M246, M545, M245, M941, M489, M609, M796, M818, I674, M179, M350, M255, I272, M402, I278, M464, M358, M000, M064, M256, M301, M622, M415, M051, M414, M354, M948, M253, M898, M317, M502, M608, M050, M053, M060, M541, M843, M351, M503, M352, M161, M868, M509, M664, M471, I6399, M611, M865, M861
**Neoplasm**	1890, 1749, 1410, 2028, 1629, 1552, 1469, 1539, 1830, 1919, 1519, 2050, 1479, 1918, 1909, 1715, 1509, 2001, 1820, 185, 1889, 1520, 1579, 1560, 2080, 1561, 2030, 1536, 1809, 1540, 1562, 1533, 1940, 1419, 1541, 2384, 2252, 1623, 2041, 1570, 1709, 1729, 1619, 2051, 1569, 1913, 1921, 1611, 2019, 1550, 1641, 2396, 1725, 1551, 2355, 1529, 2089, 1603, 1429, 1481, 1420, 2387, 1534, 1459, 1910, 1892, 1589, 1571, 1480, 1463, 1515, 1719, 1891, 1409, 1869, 2029, 2113, 2280, 1949, 1531, 2059, 2049, 226, 1874, 1739, 1510, 2040, 1625, 193, 2008, 1832, 1639, 1716, 2088, 1450, 175, 1513, 1430, 5699, 1537, 1929, 1535, 1737, 2127, 5698, 1916, 1736, 1489, 1532, 1733, 1912, 1917, 1580, 1628, 2391, 1439, 2070, 2250, 1911, 2273, 2353, 1543, 1701, 1455, 5690, 1449, 1844, 1521, 1504, 1548, 1456, 1460, 1640, 2060, 1727, 1700, 1530, 2390, 2386, 1620, 2024, 1713, 1732, 1453, 1707, 1505, 2069, 2395, 1588, 2398, 1893, 1542, 1602, 1522, 1901, 1922, 2002, 1840, 2230, 1649, 1923, 1734, 1714, 1503, 1703, 1948, 1704, 1609, 1605, 1572, 2020, 1726, 2389, 1941, 2081, 2399, 1612, 1899, 1849, 1706, 1946, 1416, 2337, 2352, 1600, 1735,	C349, C64, C845, C80, C189, C719, C509, C838, C169, C911, C61, D443, C411, C56, C259, C679, C220, D480, D469, C260, C959, C787, C920, C55, C519, C541, C109, C97, D430, C249, C159, C179, C20, C696, C329, C530, C439, C443, C241, D489, C069, C180, C859, C186, D471, C900, D329, D479, K631, C269, C539, C929, C950, C23, C73, C449, C759, C412, C459, C437, C66, C689, C343, C187, C229, D432, C482, D374, C677, C762, C780, C749, C119, C029, D381, C851, C444, C221, C419, C493, C798, C481, C499, C930, C492, C450, D65, C760, C951, C795, C320, C099, C240, C710, C718, C182, D431, C436, C819, C140, C919, C763, C833, D380, C170, C250, C07, C921, D444, C051, C910, C050, C19, C793, C383, C835, C160, C319, C341, D414, C844, C480, D352, C380, C880, C830, C831, C821, C717, C786, C901, K639, C699, C570, C139, C414, D45, C340, C716, C080, D371, C210, C254, C712, C457, D320, C927, D410, C12, C184, C925, C52, C579, C711, C820, D376, C857, C321, C447, C729, D467, C913, C039, C181, D472, C839, C709, D487,

	1800, 1574, 1421, 1702, 2000, 1740, 2397, 2357, 1622, 1915, 1601, 1741, 1512, 2023, 2072, 1538, 1920, 2375, 1838, 2061, 1914, 1943, 2118, 1511, 2126, 1440, 2392, 2021, 1885, 1880, 1610, 1471, 1906, 2132, 2212, 2078, 1728, 2251, 1724, 2115, 1730, 1500, 1452, 1879, 1900, 2388, 2394, 1624, 1400, 2111, 1710, 1604, 2031, 2270, 2377, 2117, 1613, 2038, 2299, 1712, 1454, 2385, 2380, 2116, 2381, 1748, 2370, 1887, 1907, 1718, 2364, 1462, 2354	C494, C924, C840, C148, C33, C059, D375, D383, D139, D151, C947, C062, C767, D448, C402, C837, C400, C969, C111, C211, C902, C049, C172, C629, C435, C931, D369, D464, D439, C761, C751, C009, C185, C609, C268, C834, C089, D137, C451, K630, C01, D379, C445, C060, C65, C223, C957, D157, C37, C384, C829, C680, D429, C914, C310, C792, C549, C490, K632, C713, C410, C150, C433, C788, C153, K638, C724, D481, C781, C261, D377, C694, C030, C740, K634, C782, C342, C434, C155, D333, K633, C794, C495, C171, C442, D485, C479, C311, D332, C939, K635, C940, D445, C510, C218, D477, D300, C882, C399, D440, C850, C300, D370, D412, D337, C469, D143, C154, D483, C720, C323, C741, D136, D391, C312, C313, C700, C491, D434, D449, C922, C715, C779, C068, C632, C915, C693, C472, C688, D131, D390, C161, D361, D180, C052, D350, C252, C130, D164, D126, D385, C183, C923, D174, C692, C446, C090, D372, C431, C432, C768, C765, C753, C475, C961, C100, K621, C722, C409, C301, C163, C498, C500, C440, D140, D239, C714, C578, D463, C257, C639, C967, C755, D303, C917, C251, D321, C945, C471, C430, D330, C470, C460, C809, C800, C473, C222, D165, D447, C690, D331, D473, C152, D181, C164, C188, C258, D27, C695, C413, D474, C501, C248, C962, C966, C827, C031, C577, C852, D134, C944, C631, C750

**Neurological**	3320, 3459, 3319, 3310, 2901, 3352, 2900, 3591, 3570, 3453, 3349, 3451, 3599, 3334, 3498, 3318, 3336, 3589, 3342, 3594, 3314, 3580, 3330, 2902, 3568, 3311, 3360, 3569, 3343, 2904, 3308, 3369, 3592, 2949, 3351, 2940, 3338, 3300, 3598, 3533, 3368, 3348, 3410, 3579, 3340, 3321, 2903, 3350, 3419, 3341, 3361, 3418, 3301, 3588, 3339, 3331, 3359, 3590, 3335	G20, G122, G309, F03, G409, G950, F329, F204, G10, G610, G301, G35, F059, G403, G319, F069, F179, G120, G700, G903, G039, G629, G710, F205, G060, G111, G419, G318, G709, M332, G951, F209, G239, G379, G711, F340, G713, G244, G253, G728, G959, G406, M331, G238, G311, G628, G230, G308, G908, F319, G119, G129, G418, G729, G712, F259, F322, G609, G062, G128, G373, G300, G600, F051, G249, F39, F202, G114, F220, G231, G901, G255, F29, G405, F200, G241, G232, F339, G259, G404, F323, G958, G121, G618, G061, G909, G608, F320, G030, F321, G603, G719, F171, G360, G372, G900, G402, G041, G724, G904, G258, F333, G233, F067, F172
**Neglected Tropical Diseases**	7159, 7153, 7199, 7191, 7158, 7183, 7150, 7184, 7179	B787, A090, B789, B602
**Nutrition**	2639, 2699, 7169, 261, 2810, 2818, 262, 2819, 2638, 7165, 2651, 2698, 260, 2812	E149, E145, E141, E147, E140, E142, E139, E148, E639, E146, D500, M109, E144, D539, D508, D509, E110, D510, E538, E060, E143, M100
**Other non-communicable diseases**	5997, 2898, 2500, 2733, 2720, 586, 5960, 7597, 7421, 5789, 2859, 2509, 7980, 5908, 570, 2850, 2501, 5990, 2765, 2503, 7467, 7422, 2502, 7451, 5522, 2849, 7479, 5849, 5901, 7471, 7469, 2848, 2506, 5939, 5938, 2773, 6019, 7855, 600, 2510, 2507, 7473, 5900, 2779, 2788, 5198, 5996, 7506, 2738, 2449, 7566, 7598, 2724, 2722, 6188, 7470, 7561, 5780, 5951, 2776, 2780, 7485, 2767, 2753, 2592, 7423, 2588, 2899, 2769, 7803, 2771, 7548, 7455, 5961, 2793, 2730, 2873, 6029, 2829, 2858, 7419, 2762, 2558, 6144, 7460, 5190, 7478, 2875, 7490, 2505,	E725, E46, D649, E789, Q249, E872, N319, Q248, N390, R54, D759, E059, R092, E781, E039, E86, E780, G934, N40, R99, Q823, E878, E870, D689, D693, E640, Q031, Q909, Q871, E835, E079, Q000, D619, G931, R95, E871, G919, Q897, E752, Q282, Q336, Q850, Q049, E271, N492, Q255, R53, N049, N289, E830, Q231, N302, Q874, R568, E785, E049, Q213, E668, Q446, E880, N308, Q203, E782, G98, N12, E849, Q054, N200, K430, E854, N309, K439, E875, E889, E859, D696, Q761, E840, D591, D721,

	2468, 7456, 7893, 7480, 7400, 2504, 2532, 2740, 2760, 5959, 2761, 2824, 7452, 591, 7582, 5934, 7454, 2530, 2880, 7512, 7464, 7527, 5700, 2774, 2554, 5929, 7565, 7580, 7568, 7854, 2536, 2866, 7515, 7860, 7429, 2750, 5199, 7468, 7504, 7410, 5920, 280, 2429, 7832, 6238, 7585, 2707, 587, 2770, 7450, 2879, 2775, 2512, 6021, 7424, 7453, 2869, 2826, 5952, 2772, 7581, 7583, 2778, 2420, 6088, 5881, 2515, 7586, 7558, 2706, 2831, 7847, 2839, 7511, 7599, 7830, 7474, 2732, 7462, 2520, 2535, 7476, 2763, 2874, 2820, 2728, 2895, 7513, 2830, 2766, 2550, 6191, 5969, 7483, 2792, 7589, 7863, 2511, 7484, 7472, 6012, 2832, 2754, 7463, 2700, 7567, 2159, 214, 7594, 2752, 2749, 2768, 6180, 5909, 7899, 7466, 2794, 2594, 5968, 7569, 7461, 7542, 7486, 2729, 7981, 5845, 2409, 7564, 2469, 5966, 7802, 2725, 2731, 7833, 2727, 2799, 2888, 6273, 5902, 2363, 6110, 7800, 2867, 7555, 2871, 5964, 2883, 7590, 7539, 7807, 7989, 7420, 5642, 7520, 7596, 2878, 7987, 5696, 6209, 7514, 6010, 5999, 2894, 6271, 2741, 5989, 2552, 2791, 2549, 6181, 2872	Q273, I951, Q917, Q913, Q330, E162, D589, N159, E213, R579, N320, N151, G939, Q210, D688, R960, G912, Q042, Q234, N251, E274, J701, G936, G910, G930, Q798, Q338, Q602, E831, Q610, K047, E230, N760, D735, Q212, Q246, D66, Q211, E669, E063, R090, N321, N288, E222, E853, E279, N312, E778, G09, E763, G918, N280, E048, G938, Q790, N324, D571, E858, D758, D70, Q283, Q872, Q780, E15, D609, E750, E662, D643, Q043, E41, K627, N498, Q214, E728, E876, Q224, E724, G935, Q263, N028, Q858, N930, R58, R570, E340, E888, G250, Q046, N329, Q245, N039, D569, Q938, E721, E660, Q394, D694, D739, N26, N950, Q239, N811, D752, E249, E43, E743, Q040, E751, Q251, G92, D648, Q914, Q039, Q792, Q600, R55, E762, D682, Q059, N814, N209, Q688, Q789, Q079, E232, D581, D690, Q929, Q984, D730, Q891, K431, Q244, Q998, Q899, Q459, E877, N10, Q791, Q939, Q764, Q999, E329, Q205, Q793, Q434, Q985, D699, Q990, Q675, R91, Q233, E788, N939, Q438, E841, Q250, D868, N202, N210, N201, Q928, E712, Q851, E348, Q642, Q321, Q822, E722, Q225, E713, Q795, Q878, D760, Q230, Q310, Q048, N328, Q02, D71, Q070, E050, Q809, R578, N832, D899, Q240, G961, Q870, Q208, Q771, N459, M879, E756, N142, D610, Q219, N839, G911, Q280, Q262, N141, Q315, D695, E035, N119, D561, Q319, E852, E740, D560, I959, Q201, E260, Q601, E711, Q442, E848, D562, D686, R098, D259, D761, Q204, N816, Q898, Q030,

		Q269, E833, N813, Q433, D685, D892, N281, Q605, Q606, E744, E610, Q910, Q339, D728, Q253, D762, D731, Q782, N450, N111, Q200, E164, N258, N952, N920, D684, N031, Q411, Q934, Q268, E770, Q410, D680, G968, Q630, E802, N304, E272, G932, R599, Q412, Q332, E742, Q641, Q758, Q264, E269, E723, Q218, Q348, Q676, Q752, Q044, I090, G2000, Q445, E236, E754, Q796, N399, N908, Q893, N809, Q648, N800, K049, N948, N140, E790, Q890, Q820
**Respiratory**	496, 492, 4912, 515, 5070, 4939, 5188, 4919, 5185, 4739, 502, 490, 494, 5130, 501, 5163, 505, 3249, 5109, 514, 5119, 5168, 5100, 4787, 3240, 4910, 326, 4782, 4931, 5183, 135, 5180, 504, 5110, 5118, 500, 4918, 512, 4930, 3241, 4950, 5191, 503, 4783, 5189, 5181, 5080, 5184, 4959, 5169, 5064, 5194, 5060, 5193	J449, J448, J841, J439, J81, J459, J440, J849, J80, J840, J869, D869, J852, G473, J46, J398, J390, J850, J700, J441, J679, J628, J392, J860, J387, J82, J684, J851, J348, J329, G470, J385, J848, J450, J432, J323, J386, J391, J451, J383, J430, G478, J678
**Respiratory Infection**	486, 340, 485, 1991, 179, 4870, 4871, 1490, 4809, 481, 1125, 0116, 1599, 1590, 0119, 7310, 4660, 1129, 4824, 0189, 7373, 1173, 7383, 1370, 1952, 1950, 1179, 1951, 4659, 1953, 0170, 4829, 4661, 4800, 0112, 7302, 463, 4821, 7371, 0118, 138, 4878, 4643, 1958, 0114, 3829, 4823, 4828, 130, 1128, 014, 1120, 1124, 0115, 1962, 1598, 1498, 483, 0157, 0120, 4640, 0129, 1175, 4879, 1955, 1159, 0150, 1968, 1591, 4822, 1177, 4808	J110, J47, J189, J90, J988, J159, J969, J690, J111, J209, J180, J984, J157, J40, B99, J101, A402, J100, J42, J939, J22, J61, A403, A162, J960, B909, J13, J989, A199, J118, A169, K659, J069, P023, J181, K658, J182, N709, P030, B908, J219, J150, J129, J620, A180, A409, J981, J941, J152, J188, K650, J380, J942, A400, J154, A483, J151, J986, A183, J980, J128, J985, A014, J156, A181, J041, J949, J948, J64, J120, J14, A480, A488, A401, J938, J920, A182, J961, J155, J051, J121, J09, A188, J029, A408, J210, H709, H700, A170, J982, A481, H701, J122, P234, J123, J158, A151, B900, P236, J931, J108, J040, J208, H729
**Skin**	7070, 6823, 6826, 6822, 6829, 6945, 6944, 7079, 6869, 7071, 6951, 6827,	D849, L89, L039, L031, L120, L97, L089, L024, L984, L511, L022, D860,

	7078, 6929, 6930, 6961, 6825, 4572, 6960	M725, L028, L129, L109, M726, L26, L032, L539, I898, D819, I890, L988, L309, L088, L989, L512, D831, L021, L950, L033, L048, D801, L023, D839, L899, A046, L138, D811, L893, D800, D803, L029, L038, L405, L982, L82, L409, L439, L279, L22
**Substance Use**	3040, 303, 7999, 7991, 2989, 3049, 3050, 3059, 2982, 3051, 7993, 2912, 2918, 3042, 2960, 7994, 2956, 2959, 3055, 7990, 3559, 2965, 2910, 3004, 7998, 2919, 2953, 2961, 3056, 2911, 3000, 7907, 3019, 2952, 7908, 3525, 3009, 2966, 2971, 2969, 7906	F102, F019, F101, F103, G312, F113, F191, G310, F100, F107, F112, F104, F109, F011, Y10, F149, R060, P960, F199, F150, R048, R040, F192, F140, F193, F111, F106, R001, F141, F119, R02, F159, F151, Y15, F018, P044, F013, R75, F010, R042, F012, F131
**Unintentional**	E8601, E8191, E9871, E8190, E8147, 3430, E8500, E885, E8609, E8129, E8788, E8842, E9192, E8121, E911, E888, E887, E8120, E9104, E9138, E9010, E882, E8159, E8589, E8938, E8552, E9421, E8902, 3429, E8199, E9019, E8588, E9139, E9240, E9109, E8227, E8136, E8161, E8152, E8785, E916, E9290, E8160, E8809, 3439, E9872, E8532, E8101, E8849, E8781, 3448, E8784, E8912, E8169, E9299, E8682, E9800, E8122, 3483, E8150, E8700, 3481, E9108, E984, E8232, E851, 3449, E8501, E9478, E9889, E9102, E8162, E8939, E8580, E8798, E9103, E8585, E9259, E8624, E8192, E8789, E8189, E8551, E8550, E8196, E8690, E9289, E8841, E8520, E8810, E8540, E8252, E9293, E8799, 3485, E8508, E9043, E9041, E9854, 3489, E9179, E9068, E8903, E9345, 3484, E912, E8581, E8123, 3440, E8786, 3441, E9350, E8603, E8583, E8860, 3488, E9197, E8502, E9479, E8768, E9805, E8706, E8782, E9101, E8261, E9232, E8704, E8908, E8743, E8181, E8211, E899, E8210, E9288, E9130, E8193, E8586, E9803, 3482, 3499, E9229, E927, E8602, E8200, E9131, E8156, E8783, E9022, E8282, E9880,	W78, X59, W19, X42, X64, X61, W22, X44, X81, X95, W01, X99, X48, X67, X62, Y839, X93, W80, W74, W69, Y450, W70, W17, X09, X82, W06, X31, X41, W16, W31, X00, X45, W84, W08, Y831, X66, W10, X63, W94, W11, W79, X46, X91, W20, Y848, W34, W03, W55, W65, Y600, W24, X83, W73, X40, X94, W13, X84, X47, Y832, Y834, X11, W83, X85, W23, X53, W15, Y520, W02, X15, W18, X69, W49, X49, Y838, W05, W66, Y846, X39, Y495, W12, W00, X68, W75, W86, X92, X65, X30, W67, Y833, W07, W92, X08, W76, W68, X97, X23, X88, W81, X36, X58, W25, W85, Y579, Y835, W26, Y420, W14, X43, Y433, W50, X32, X04, Y881, W77, W29, W36, W54, X05, W51, Y836, Y453, Y883, W44, X02, Y445, Y601, Y442, X590, X599, Y844, Y401, Y604, Y411, Y466, X03, Y880, Y830, Y409, Y443, Y434, W04, X06, Y821, Y575, X01, Y658, Y788, Y841, W27, Y462, W40, L509, Y791, W46, W45, Y400

	E909, E8556, E9870, E9100, E9879, E8839, E8811, E9228, E9258, E9193, E9198, E9050, E8151, E9457, E8153, 3442, E9132, E9170, E9352, E9320, E8763, E9208, E8100, E9199, E9342, E8130, E8531, E8699, E8528, E9190, E8226, E8911, E8126, E9354, E8683, E9804, E8796, E9194, E8111, 3432, E8621, E8780, E9830, E8505, E9351, E9191, E9379, E8840, E8582, E8688, E892, E9384, E9838, E9460, E8689, E9363, E9133, E8790, E8931, E8529, E9839, E8250, E8179, E9321, E8909, E8830, E9809, E9238, E8800, E8163, E8705, E8981, E9209, E9420, E8930, E8132, E9447, E9831, E8708, E918, E9251, E9881, E9308, E8538, E8259, E8542, E9885, E8220, E8692, E9358, E9398, E8131, 3446, E8197, E8869, E9473, E9390, E8149, E9331, E9309, E8148, E9018, E8769, E8212, E8641, E8668, E9802, E915, E8530, E986, E8182, E9009, E8127, E8230, E8680, E9499, E9292, E8229, 3438, E9888

Subset Cause ICD 9 and 10 Codes:

CAUSES	ICD9	ICD10
**Lung**	162.9	C349
**Prostate**	185	C61
**Breast**	174.9	C509
**Colon**	153.9	C189
**Pancreas**	157.9	C259
**Esophagus**	150.9	C159
**Ovary**	183.0	C56
**Ischemic Heart Disease**	410-414	I20-25
**Stroke**	430-438	I60-I69

Results of the sensitivity analyses after reassignment of residential CTs

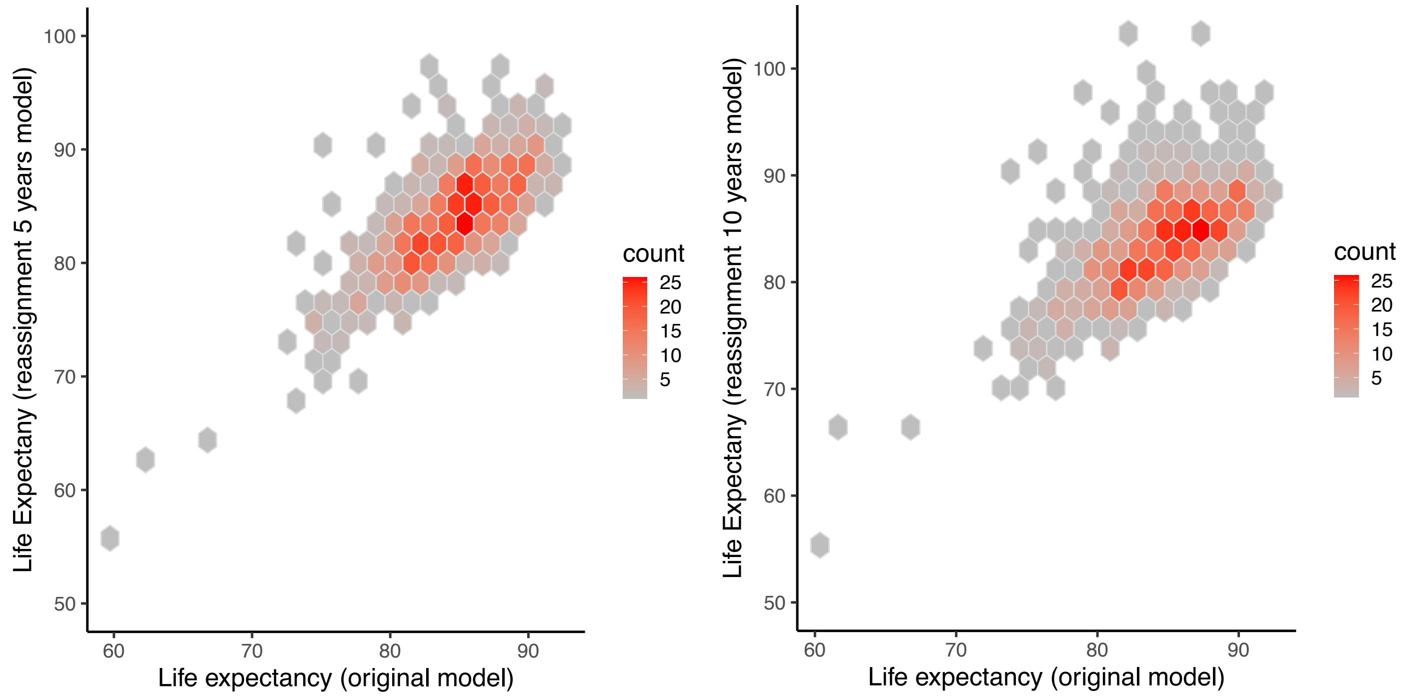

Hexbin density plots analysis: There were more data points and agreement of CTs with life expectancy estimates between 80-90 years old. Although there are less data points for LE estimates of <75 years, the overall trend still follows a linear pattern, meaning there was not a large change between the reassignment and original models even for more extreme estimates. In the 10-year reassignment model, the spread of the data points is greater, meaning there were less agreement and more impacts of migration on the LE estimates. This is not surprising given the assessment of a longer time period of residence, and therefore a greater chance for migration to occur or the assignment of another CT using duration of residence.
